# The impact of life tables on age standardized net survival of real-life example databases

**DOI:** 10.1186/s12874-025-02600-7

**Published:** 2025-05-26

**Authors:** András Wéber, Zoltán Vokó, Zoltán Kiss, István Szatmári, Mária Dobozi, Petra Parrag, Ibolya Fábián, György Rokszin, Péter Nagy, Csaba Polgár, István Kenessey

**Affiliations:** 1 National Institute of Oncology and National Tumor Biology Laboratory, Budapest, Hungary; 2 Center for Health Technology Assessment, Semmelweis University, Budapest, Hungary; 3 Center for Pharmacology and Drug Research & Development, Semmelweis University, Budapest, Hungary; 4 Syreon Research Institute, Budapest, Hungary; 5 Second Department of Medicine and Nephrology-Diabetes Centre, University of Pécs Medical School, Pécs, Hungary; 6 Schools of PhD Studies, Semmelweis University, Budapest, Hungary; 7 RxTarget Ltd., Szolnok, Hungary; 8 University of Veterinary Medicine Budapest, Budapest, Hungary; 9 Department of Anatomy and Histology, HUN-REN–UVMB Laboratory of Redox Biology Research Group, University of Veterinary Medicine, Budapest, Hungary; 10 Chemistry Coordination Institute, University of Debrecen, Debrecen, Hungary; 11 Department of Oncology, Semmelweis University, Budapest, Hungary; 12 Department of Pathology, Forensic and Insurance Medicine, Semmelweis University, Budapest, Hungary

**Keywords:** Cancer survival, Cancer epidemiology

## Abstract

**Background:**

Population-based, age-standardized net survival estimates provide valuable insights for comparing the effectiveness of cancer treatment and the prospects of cure in an international context. Although numerous studies have previously assessed survival, the choice of life tables may crucially impact the feasibility of such analyses. Therefore, based on available studies, our aim was to understand the critical influence of life tables on net survival estimates.

**Methods:**

Record-level data of approximately 50,000 breast, cervical, and ovarian cancer patients were extracted from the Hungarian National Cancer Registry. These patients were diagnosed between 2010 and 2014 and were followed up until December 31, 2019. Life tables for the Hungarian female population were taken from the Human Mortality Database, the Human Life-Table Database and were compiled according to the EUROCARE, CONCORD both multivariable flexible and Ewbank methodology. Regarding the last due to the lack of specific parameters, simulations were performed to assess the missing values​. The calculation of 5-year age-standardized net survival using different life tables revealed limitations in the methodology, highlighting the impact of life table selection on survival estimates.

**Findings:**

Minor biases were observed in age-standardized net survival when using life tables from different international databases. However, the net survival of breast cancer, which had the most favorable prognosis of the studied malignancies, showed significant discrepancies. Moreover, this research highlights the extreme sensitivity of the applied κ parameter in the CONCORD Ewbank method, underscoring the need for careful consideration when applying this approach.

**Interpretation:**

Present study shed light on how the choice of life tables can lead to differences in survival estimates for the same cancer population. It also emphasizes the importance of open methodological discussions to improve validity and accuracy of international comparability.

**Supplementary Information:**

The online version contains supplementary material available at 10.1186/s12874-025-02600-7.

## Introduction

Population-based cancer survival analyses are essential for assessing, developing and refining nationwide cancer control plans. They also provide valuable insights into the quality of clinical care, cancer patient management, and the impact of developing treatments and therapies. Variations in these survival indicators across countries offer crucial information for healthcare professionals and policy makers, placing them in the forefront of interest.

Extensive collaborations have sought to analyze these survival trends across different geographic regions and time periods. The International Agency for Research on Cancer’s Global Cancer Observatory (IARC’s GLOBOCAN) periodically shares survival data [1]. Within this framework, SURVMARK-2 project provides comprehensive population-based cancer survival data for selected high-income countries, while SURVCAN-3 primarily focuses on lower-income settings [2, 3]. Another comprehensive initiative, EUROCARE-5, monitored cancer survival for patients across nearly 30 European countries, covering the ten most frequent cancers [4]. Recently, successive waves of the CONCORD research program have reported on global cancer survival, based on data from population-based cancer registries [5, 6]. To estimate cancer survival, the aforementioned studies applied similar methodologies. Specifically, they used background mortality data to calculate 5-year net survival using the Pohar Perme method [7].

While these studies are methodologically well-founded, their results can be subject to strong and legitimate criticism. Differences in local coding and classification systems, diverging cancer registration practices, and data quality issues across countries pose potential challenges to international comparisons of population-based cancer survival [2]. In addition, different studies have used life tables from various sources, leading to at least minor discrepancies in background mortality, which may impact estimated outcome and overall comparability.

This study aims to contribute to the ongoing methodological discussions by examining in detail how different life tables (LT), functioning as background mortality, influence cancer survival. Our research is based on follow-up data from Hungarian female breast, cervical and ovarian cancer population registered between 2010 and 2014, sourced from the population-based real-world data of the Hungarian National Cancer Registry (HNCR). From these, 5-year age-standardized Pohar Perme net survival probability were calculated using the most commonly applied LTs, including the CONCORD multivariable flexible model (CONCORD-MFM), the EUROCARE, the Human Life-Table Database (HLD), and the Human Mortality database (HMD) [8, 9, 10, 11], calculated for the Hungarian population. Exception was the CONCORD Ewbank relational model with various parameters (CONCORD-EWBANK), where the survival could not be determined due to the lack of specified parameters (κ, λ). Consequently, simulations were performed with gradually varying values. Thus, in this case our combination of parameters multiplied the obtained survival probabilities.

## Materials and methods

### Data sources

Hungarian female death counts by single ages (0–99 years) for the calendar years 2009–2020 were extracted from the Eurostat database, which is compiled based on the mortality reports of the Hungarian Central Statistical Office (HCSO) [12, 13]. However, single-age mortality data in the Eurostat database were incomplete for the examined period: data were available only up to age 85 for 2009–2011, up to age 90 for 2012–2013, and up to age 100 thereafter. To ensure consistency, person-years at risk were obtained from the Human Mortality database (HMD). Previously prepared complete period life tables, used to calculate net survivals, were extracted from HMD and Human Life-Table (HLD) databases [10, 11]. The source of the HLD are the HCSO life tables, for this reason the two databases are identical. Record-level follow-up data for patients registered between 2010 and 2014 with malignant neoplasms of female breast (C50), cervix (C53), and ovary (C56) were obtained from the Hungarian National Cancer Registry (HNCR). Patients were categorized by single age, and for each the exact date of death was recorded until 31 December 2019 [14]. Vital status in the HNCR was originated from quarterly reports of the National Health Insurance Fund of Hungary (NHIF), which covers the whole domestic population [15]. Basic quality control procedures were applied, including an assessment of the morphologically verified rate (MV%) and the rate of death certificate-only cases (DCO) [15, 16]. Former represents the percentage of tumors confirmed by histological examination, while the latter reflects the proportion of cancers for which no information other than the death certificate stating the underlying cause of death can be obtained. In addition, cases with negative survival duration date (where death occurred before diagnosis) were excluded from further analysis. Additionally, cases reported solely from death certificates were not considered due to lack of survival time information.

## Life table preparation

Regarding methodology, the CONCORD Program used two approaches for the calculation of life tables across the examined countries: firstly the *‘Multivariable flexible model’* and secondly the *‘Ewbank relational model with parameters’* [8].

The *‘Multivariable flexible model’* was applied to derive the required age- and sex specific mortality rates. For this, data were obtained from the Eurostat database and the HMD: death and population count for three calendar years surrounding a central year to prevent year-to-year fluctuations. Then, the data were modelled separately for each calendar year within the generalized linear model framework, using a Poisson error and log link function, along with a restricted cubic spline function for age [8]. This model was implemented using splines package in R [17]. Splines are set up of piecewise polynomial functions joined at age-positions so-called knots. In the works of Spika et al. and Rachet et al. for male population five knots were fixed based on the age-specific death rates and a priori knowledge. In the beginning age 0, 1, and 2 where age-specific mortality changes rapidly in early life, secondly age 50 from which mortality fluctuations are reduced and thirdly age 88, which was the median age of the population who belonged to the last examined age group. Then, using simulation, three points were determined between 2 and 50 years to catch the various inflexion points observed in the mortality rate: they were given to ages of 14, 15 and 27. In summary, the following knots were used for the male population: age 0, 1, 2, 14, 15, 27, 50 and 88. The exact placement of knots were not defined for women in their study [8, 18]. In our examination eight knot locations were applied at ages 0, 1, 2, 10, 20, 35, 50 and 95 which generally better characterizes the age profile of female mortality. Four locations at ages 0, 1, 2, 50 were left unchanged due to basic human mortality dynamics. Other four knots were changed (from 14 to 10, from 15 to 20, from 27 to 35 and from 88 to 95) considering the differences in the young adult excess mortality hump [19] between males and females, which has a significantly milder course for the latter, as well as the substantially longer average life expectancy of women than men in Hungary [20, 21].

The *‘Ewbank relational model with parameters’* is a reducible four-parameter system for modelling life tables. This method utilizes unsmoothed mortality rates in the same single age breakdown to derive a smoothed mortality profile for the given population [22]. This approach is originally based on the Brass relational logit model [23], which plots the linear relationship between the logits of the standard and observed survivorship functions, the lx columns of life tables. This procedure creates two parameters according to the methodology of linear regression: α which describes the level of mortality, and β which characterizes the strength of the relationship between the standard and observed data. These parameters are then used to smooth the observed survival function. Further fitting is done by the Ewbank method by adding two additional parameters: κ for younger ages, and λ for older ages, separated by the median age of the death distribution [8]. As with the CONCORD model, the 2010 Germany CONCORD life tables for females were used as the standard [24].

Additional period life tables were constructed according to the EUROCARE methodology for the Hungarian female population. In this case a standard exponential method was used to calculate death probabilities from the mortality rates. This procedure supposes that survival time is exponential, and that in any one-time interval the death rate is constant [9]. We also used data from the HMD and HLD databases without changes for 2010–2019 as background mortality. In the HMD, the life tables uniquely do not extent to 100, but to age 110, whereas those from the HLD are identical to those of the Hungarian Central Statistical Office (HCSO) [10, 11, 25]. For the oldest age group, we excluded individuals who turned 100 during the observation interval (HLD, CONCORD-MFM, EUROCARE), with the exception of HMD, where all followed cases fit within the 110-year age limit.

## Survival analysis

To assess population-based cancer survival, 5-year overall survivals rates were calculated for malignant tumors of female breast (C50), cervix (C53), and ovary (C56), based on the 10th Revision of International Classification of Diseases. This indicator shows the proportion of patients who are still alive five years after their diagnosis with cancer, regardless of the cause of death, assuming that no cancer cases are lost during follow-up, as HNCR receive data on the event of death from multiple national population-based data sources. Survival intervals were defined as the period from diagnosis to death or to the last follow-up date. Estimated net survivals were calculated using the Pohar Perme method, which represents the survival of patients in a defined resident population, assuming cancer is the only possible cause of death [7]. Survival parameters were accompanied by 95% CIs to ensure fair comparisons across groups with varying risks of death form other causes. Survival estimates for 5 years after diagnosis were calculated for distinct age groups, with standardization based on the international cancer survival standards [26]. All calculations were carried out using the *relsurv* package [27] in **R** [28].

## Sensitivity analysis

Simulations were performed by gradually adjusting the Ewbank relational model parameters, κ and λ, and monitoring how the 5-year age standardized Pohar Perme net survival probabilities changed as a result. To determine the parameter values, we relied on the intervals found in the Ewbank-study: -0.7 to 0.7 for κ and − 0.5 to 0.5 for λ [22]. Subsequently, we fixed one parameter at reasonable values (κ = 0.05 and λ = 0.4) and changed the opposite parameter on a given scale accordingly: -0.5 < λ < +0.5 and 0 < κ < 0.07. We displayed our results in coordinate systems where the vertical axis represents the calculated 5-year age standardized Pohar Perme net survival probabilities and the horizontal axis demonstrates the values of the Ewbank parameters κ and λ. During the examination, we graphically displayed constant survival values calculated using other types of life tables, including HLD, HMD, EUROCARE, and CONCORD multivariable flexible model. The estimated interval of parameter κ was determined by empirical testing. In a way that, where the CONCORD-EWBANK curve intersects the lowest and highest constant survival represents the lower and higher bound of the κ parameter estimation on Fig. 2.

## Results

Between 2010 and 2014, breast cancer accounted for the highest number of cases among the selected tumors, with a total of 37,858 cases registered in the HNCR. Of these, 10,679 patients died during the 5-year follow-up period. This patient group had a median age of 63 at diagnosis and the highest median age at death (72 years), resulting in the highest 5-year overall survival of 71.8%. Of the tumors examined, basic quality control measures were the most favorable for breast cancer, with the highest morphologically verified rate (MV%) of 72.8% and the lowest death certificate-only rate (DCO%) at 1.1%. Cervical tumors were the least frequently reported with 5,369 cases, of which 2,485 resulted in death. This group had the youngest median at diagnosis (55 years) and at death (62 years), leading to a 5-year survival probability slightly above 50% and exhibiting slightly lower data quality indicators of MV% and DCO% compared with breast cancer. Ovarian cancer had a total of 6,541 recorded cases, with 4,103 deaths, showing the lowest 5-year overall survival of 37.3%. Consequently, the difference between the median age at diagnosis (64 years) and the median age at death (68 years) was the lowest among the examined malignant neoplasms, at only 4 years. At the same time, data quality measures for ovarian cancer were the lowest with an MV% of 42.9% and a DCO% of 2.8% (Table 1).

**Table 1:** Descriptive statistics of the selected gynecological cancer localizations registered by the HNCR, 2010–2014

	Total number of		Median age at		Percentage of	Quality indicator (2018) of	
	diagnoses	deaths*	diagnosis (years)	death (years)*	5-year overall survival (CI: 95%)	MV%	DCO%
Breast (C50)	37 858	10 679	63	72	71.8 *(71.3–72.2)*	72.8%	1.1%
Cervix (C53)	5 369	2 485	55	62	53.7 *(52.4–55.0)*	70.9%	1.2%
Ovary (C56)	6 541	4 103	64	68	37.3 *(36.1–38.5)*	42.9%	2.8%

*cases were followed up to 5 years

Source: HNCR and Wéber et al., 2023

There were significant age profile differences in the nature of examined gynecological tumors. Breast and ovarian tumors had the lowest proportion of registered cases in the youngest age group (15–44), at 9.7% and 11.3%, respectively, whereas cervical cancer had the highest proportion at 27.1%. Breast and ovarian cancers were most commonly diagnosed in older age groups, with 28.9% of breast cancer cases occurring in the 55–64 age group and 26.3% of ovarian cancer cases in the 65–74 age group. In the study population, 42.7% of breast cancer deaths occurred in the oldest age group (75+), while nearly 30% of ovarian cancer deaths and only 22.4% of cervical cancer deaths were from this age group. Deaths due to cervical and ovarian tumors were most frequent in the 55–64 (26.2%) and 65–74 age groups (29.7%), respectively.

The 5-year overall survival probability for breast cancer declines with age, dropping from 86.1% in age group 15–44, to 43.4% in age group 75+. For cervical and ovarian cancer, the decline is even steeper, with survival probabilities falling to approximately one-fifth of their initial values, from 77.6 to 15.5% for cervical cancer and and from 69.6 to 15% for ovarian cancer (Table 2).

**Table 2:** Descriptive statistics of the selected cancer localizations registered by the HNCR broken down by age groups, 2010–2014

	Number of		Percentage of			
	diagnoses	deaths*	diagnoses	deaths*	ICSS Standard**	5-year overall survival (CI: 95%)
**Age group (years)**	**Breast (C50)**					
15–44	3 660	510	9.7	4.8	7.0	86.1 *(85.0-87.2)*
45–54	5 944	889	15.7	8.3	12.0	85.0 *(84.1–86.0)*
55–64	10 925	2 069	28.9	19.4	23.0	81.1 *(80.3–81.8)*
65–74	9 289	2 656	24.5	24.9	29.0	71.4 *(70.5–72.3)*
75+	8 040	4 555	21.2	42.7	29.0	43.4 *(42.3–44.5)*
**Age group (years)**	**Cervix (C53)**					
15–44	1 457	326	27.1	13.1	28.0	77.6 *(75.5–79.8)*
45–54	1 127	441	21.0	17.7	17.0	60.9 *(58.1–63.8)*
55–64	1 319	651	24.6	26.2	21.0	50.7 *(48.1–53.5)*
65–74	807	510	15.0	20.5	20.0	36.8 *(33.6–40.3)*
75+	659	557	12.3	22.4	14.0	15.5 *(12.9–18.5)*
**Age group (years)**	**Ovary (C56)**					
15–44	738	224	11.3	5.5	7.0	69.6 *(66.4–73.0)*
45–54	964	473	14.7	11.5	12.0	50.9 *(47.9–54.2)*
55–64	1 709	989	26.1	24.1	23.0	42.2 *(39.9–44.6)*
65–74	1 718	1 217	26.3	29.7	29.0	29.2 *(27.1–31.4)*
75+	1 412	1 200	21.6	29.2	29.0	15.0 *(13.3–17.0)*

* cases were followed up to 5 years

** Corazziari et al., 2004

Source: HNCR

**Fig. 1:**
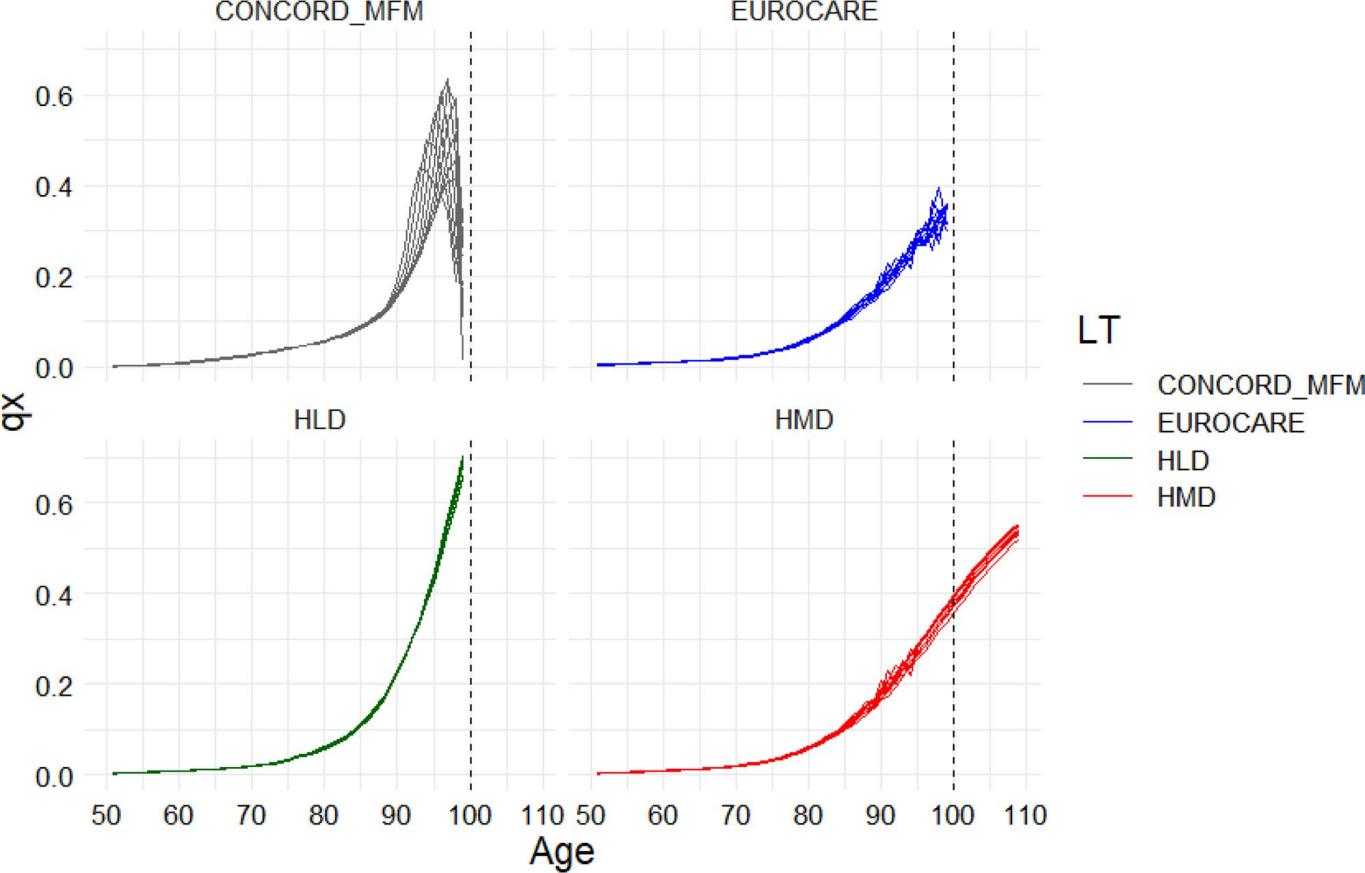
Probability of Hungarian women dying between exact ages x and x + 1 (qx column in LT) by different source of life tables, 2010–2019. Abbreviations: Human Mortality Database, HLD - Human Life-Table Database, CONCORD-MFM– CONCORD Multivariable Flexible Model. Sources: Eurostat database, Human Mortality Database, Human Life-Table Database

Qx refers to the probability of dying between exact ages x and x + 1 and is the only column in the LT used in the Pohar Perme survival calculation procedure. Among the LT varieties, HLD shows the steepest increase with age, reaching the highest values while maintaining the smallest annual variance. This is followed by the CONCORD multivariable flexible model which has a similarly high increase, but unlike HLD, it begins to decrease before the age of 100 at varying rates each year, leading to significant deviations across calendar years. Thirdly, the HMD also increases with age, though to a lesser extent and in a unique way: its values extend beyond age 100, reaching up to 110 years. Furthermore, from age of 95 onward, smoothing of qx values become apparent. Lastly, EUROCARE follows a growth pattern very similar to HMD but with larger annual deviations and a limit of 100 years of age (Fig. 1).

In our Pohar Perme estimations, we observed that HLD life tables generally yield the highest survival probabilities (except for cervical cancers), followed by HMD, the CONCORD multivariable flexible model, and lastly EUROCARE, which shows the lowest survival estimates. We found substantial difference in the case of breast cancer, where the interval was 78.33–80.81%, resulting in a difference of approximately 2.5% points. For cervical and ovarian malignant neoplasms, the survival intervals were 54.95–55.19% (difference: 0.24% points) and 37.24–37.8% (difference: 0.56% points), respectively. When excluding the HLD life tables from the comparison, the differences decrease even further: for breast cancer, the discrepancy reduces from 2.5 to 1.1% points, and for ovarian tumors from 0.56 to 0.33% points. Cervical cancers are considered an exception in this regard, as the 0.24% point difference remains unchanged due to HLD not yielding the highest value (Table 3).

**Table 3:** 5-year age standardized Pohar perme net survival probabilities in percentages with 95% confidence intervals for Hungarian women by selected gynecological cancers diagnosed in 2010–2014

Life table	Breast (C50)	Cervix (C53)	Ovary (C56)
HMD	79.35 *(78.16–80.57)*	55.19 *(52.25–58.38)*	37.57 *(34.95–40.42)*
EUROCARE	78.33 *(77.16–79.52)*	54.95 *(52.04–58.11)*	37.24 *(34.65–40.05)*
HLD	80.81 *(79.44–82.22)*	55.15 *(52.15–58.43)*	37.80 *(35.11–40.73)*
CONCORD-MFM	78.87 *(77.69–80.08)*	55.13 *(52.20-58.31)*	37.43 *(34.82–40.25)*

Abbreviations: HMD - Human Mortality Database, HLD - Human Life-Table Database, CONCORD-MFM– CONCORD Multivariable Flexible Model

Sources: Hungarian National Cancer Registry, Eurostat database, Human Mortality Database, Human Life-Table Database

**Fig. 2:**
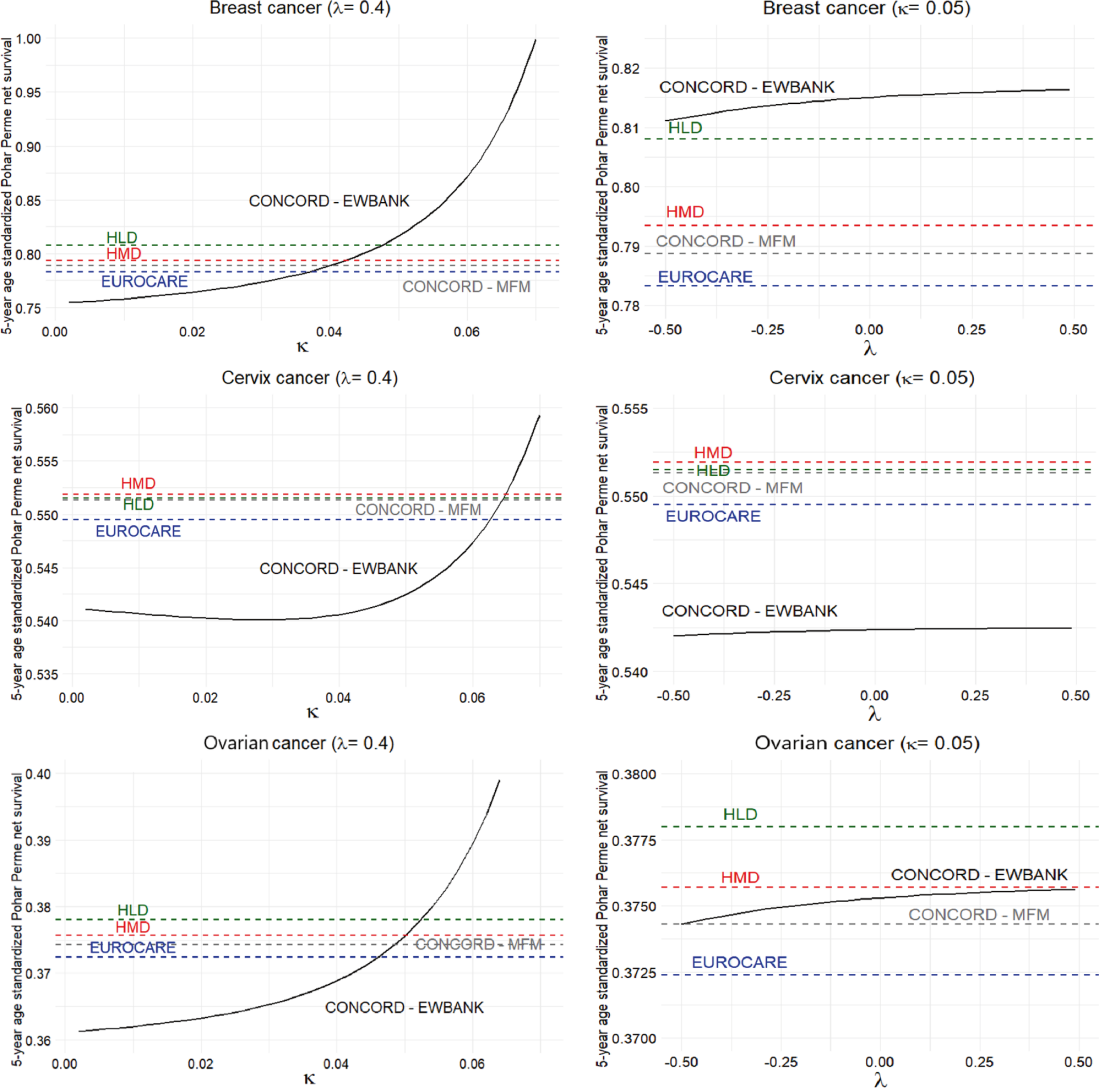
Estimated 5-year age standardized Pohar Perme net survival probability for Hungarian patients registered between 2010–2014 by different LT methods and selected gynecological cancers with varying and fixed kappa and lambda values. Abbreviations: Human Mortality Database, HLD - Human Life-Table Database, CONCORD-MFM– CONCORD Multivariable Flexible Model. Sources: Hungarian National Cancer Registry, Eurostat database, Human Mortality Database, Human Life-Table Database

If κ parameter is gradually adjusted while keeping λ fixed, the survival estimates calculated using the CONCORD-Ewbank methodology follows an exponential curve. According to our research, for breast cancer κ should be set between approximately 0.037 (intersection of CONCORD-EWBANK and EUROCARE) and 0.047 (intersection of CONCORD-EWBANK and HLD) to ensure that survival falls within the interval mentioned above. Using the same approach, the interval for κ is 0.063–0.065 for cervix and 0.046–0.053 for ovarian tumors. It must be emphasized, that if κ remains below the recommended interval, survival is significantly underestimated, whereas if it is above the interval, a substantial overestimation occurs. All of this arises from the exponential nature of the function, underscoring the high sensitivity of the κ parameter setting. In contrast, when λ is gradually adjusted while keeping κ fixed, the survival estimates calculated using the CONCORD–Ewbank methodology form a curve that is almost horizontal. This indicates that the λ parameter has minimal influence on the calculated survivals (Fig. 2).

## Discussion

Since the assessment of cancer survival is crucial for developing nationwide cancer control plans, quality assurance of diagnostic tools and therapeutic modalities, there is growing interest in international comparability. The widely accepted Pohar Perme model may provide population-based estimations through the application of life tables. Our goal was to examine in detail the impact of different LTs functioning as background mortality on cancer survival. According to our results, of the three gynecological tumors registered by the HNCR, only breast cancer showed a notable difference in net survival when using different LTs (2.5% points). Although we did not measure any other cancer types with relatively better prognoses (such as melanoma, prostate, bladder), we expect similar patterns in these cases as well, since the unique properties of life tables are likely to express more significantly, particularly through longer survival. Thus, they can manifest cumulatively condensed into survivals. During our research, we were able to identify a ranking among LTs based on the extent of their impact on survival. Accordingly, the HLD table generally provided the highest survival estimates, followed by HMD, CONCORD-MFM, and ultimately EUROCARE, which produced the lowest survivals. This trend is supported by Fig. 1, where the curve rises more sharply with age, indicating that as more people of similar age are excluded from follow-up, survival probabilities increase. This was most evident in the case of the HLD, where the highest death probability at 80 + corresponds to the lowest expected survival of cancers in the follow-up. Thus, this increases net survival the most among life tables, which implies significant overestimation. Nevertheless, this procedure employed the Böckh-method of smoothing with a quadratic function exclusively by the Hungarian Central Statistical Office and for this reason, HLD is not recommended for international comparison [29]. CONCORD-MFM framework shows realistic net survival results compared to calculations using other types of LTs. However, based on Fig. 1, criticisms also arise. In this case, qx curves are the only ones falling towards zero for ages above 95 year, which suggest that the probability to die for the 95 + is very small, which is an unrealistic scenario and probably a false result of overfitting. This may depend on the choice of the knot for the oldest population - in this case, 95 years of age similarly - but due to the likely low number of cases in this group, this did not appear to have a significant impact on survival outcomes. Considering further LTs, it is also apparent how much extending death probabilities to the age of 110 and smoothing from the age of 95 in the HMD database impacts survival. For example, this adjustment led to a 1.02% point increase for breast cancer survival compared to EUROCARE.

Based on the description of the CONCORD-3 methodology published in 2018, CONCORD researchers did not provide a precise definition of how life table methodologies were selected for each country [6]. However, it seems to have been decided based on where age-specific death and population figures or age-specific death rates were available for a specific country, as well as recommendations from local professionals. Based on the available supplemental materials, we collected which methodologies were used in European countries by CONCORD. The multivariable flexible model was applied for smoothing in the following countries: Finland, Greece, Iceland, Latvia, Lithuania, Netherlands, Portugal, Romania (Cluj), Spain, United Kingdom. The Ewbank relational model with parameters was used in Austria, Belgium, Bulgaria, Czechia, Denmark, Estonia, Germany, Ireland, Malta, Norway, Poland, Slovenia, Spain, and Sweden; and lastly CONCORD researchers relied on unique local data sources for France, Italy and Slovakia. Our results indicate that the survival estimates using the two CONCORD methodologies are identical for significantly differing κ values ​​per tumor type. If this condition is not met, even a small deviation can lead to fundamental changes in survival.

**Fig. 3:**
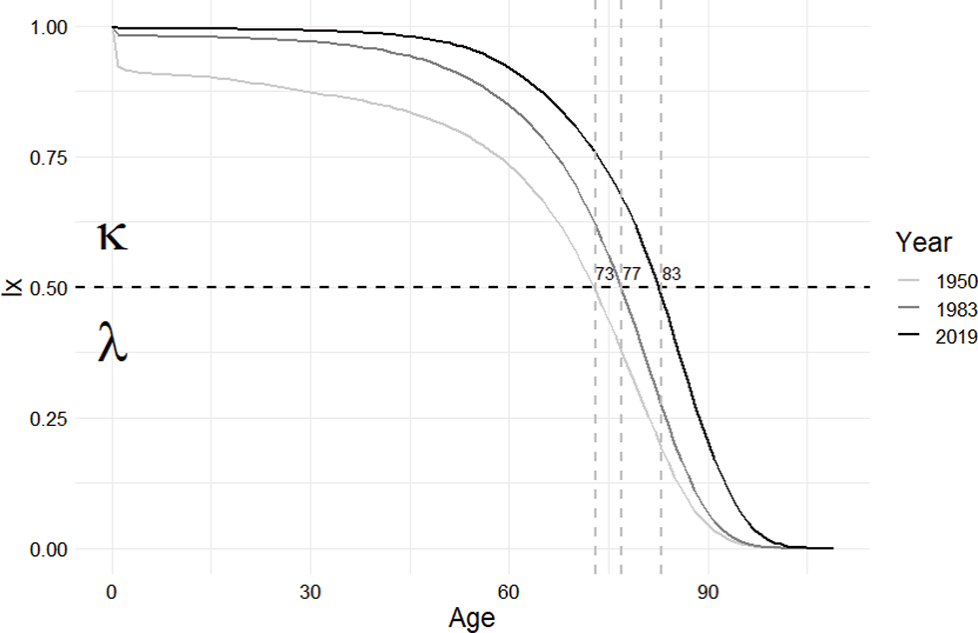
Ratio of Hungarian women alive at exact age x (lx column in LT), highlighting the median ages by year. Source: human mortality database

Consequently, it is necessary to calculate the survival of each tumor with a separate κ, which seems impractical to let the LT depend on the cancer type at first sight. However, the strength of the Ewbank methodology lies in its variable parameters, which provides flexibility for adapting it to different situations or needs. In this regard, further examinations aimed at determining parameter values minimizing the bias in survival can be way forward to ensure international comparability. It must be emphasized that since the introduction of Ewbank’s method in 1983, more than 40 years have passed, during which life expectancies in populations have substantially increased. For instance, in Hungary the median age of death distribution has risen by 6 years, from 77 to 83 [11] (Fig. 3). Considering the age distribution of cancers, this led to an imbalance between κ and λ (in favor of κ), which manifests in the former’s hypersensitivity and the latter’s insensitivity to modifications.

We must emphasize the uniqueness of this study. Through our direct and profound research, we identified only a few similar articles on this topic, most of them support our results. The first, published by Ellison, analyzed the bias in cancer survival introduced by outdated life tables and found that using historical LTs versus more recent ones may lead to consequential overestimation of cancer survival [30]. The second, by Ellis et al., examined the impact of life tables adjusted for smoking on the socio-economic differences in net survival for laryngeal and lung cancer. According to its conclusion using smoking-adjusted life tables to estimate net survival has only a small impact on the deprivation gap in survival, even when inequalities are substantial [31]. The third by Stroup et al., compared five-year relative survival using state-specific life tables to five-year relative survival using US-based life tables and assessed variations by age, race, and cancer site for all cancers combined, lung, colorectal, prostate and female breast cancers. Their results indicate, that differences between relative survival based on the two LTs were small and state-based estimates were less reliable than US-based estimates for older populations aged 85+ [32]. Finally, another study estimated the impact of social gradient on survival of people with cancer. According to their results, the use of deprivation-specific lifetables confirms the existence of a social gradient in cancer survival, indicating that these inequalities do not result from inequalities in background mortality [33]. We demonstrated that changing the different LTs -specifically HLD, HMD, EUROCARE, and CONCORD-MFM- alone did not result in major differences in tumor survival. However, the better the prognosis of a malignancy, the greater the potential survival differences. For instance, in case of breast cancer there was a 2.5% point difference (EUROCARE: 78.3% vs. HLD: 80.8%) as reported in this study. On the other hand, slight modifications to κ in the CONCORD-EWBANK method can fundamentally change the survival for the corresponding cancer. Based on the breast cancer survival data from CONCORD-3, these small but notable differences may influence the ranking of countries in international comparisons, either lowering or raising their standing among European nations [6]. Of particular note, our survival analysis of ovarian cancer, when compared with the data from Arnold et al., revealed that Hungarian net survival between 2010 and 2014 was in the same range as that of high-income countries, at ~ 37.5%, compared to 36.0-46.2% (Table 4).

**Table 4:** 5-year age standardized Pohar perme net survival probabilities in percentages with 95% confidence intervals for selected countries by ovarian cancers diagnosed in 2010–2014 from different sources and their differences

Countries	ICBP SURVMARK-2	CONCORD-3	difference (in % points)
Australia	43.2 *(41.3–45.1)*	42.0 *(40.8–43.2)*	-1.2
Canada	40.3 *(39.0-41.6)*	40.9 *(39.9–41.8)*	0.6
Denmark	42.1 *(39.8–44.5)*	39.7 *(37.8–41.6)*	-2.4
Ireland	36.0 *(33.0-38.9)*	32.8 *(30.3–35.3)*	-3.2
New Zealand	36.3 *(33.3–39.4)*	36.7 *(34.1–39.3)*	0.4
Norway	46.2 *(43.6–48.8)*	45.5 *(43.3–47.7)*	-0.7
United Kingdom	37.1 *(36.5–37.8)*	36.2 *(35.7–36.8)*	-0.9
Hungary*	37.6 (35.0-40.4)**	37.4 (34.8–40.3)***	-0.2

* Own results

** based on HMD life tables

*** based on CONCORD - Multivariable Flexible Model life tables

Survival calculation frameworks of SURVMARK-2 and CONCORD-3 differ technically in multiple ways. Firstly, the two studies applied different LTs, secondly, CONCORD-3 excluded death certificate-only cases from the calculations, while SURVMARK-2 included a 1-day follow-up for those cases. Regarding LTs, SURVMARK-2 has an advantage in survival calculations over CONCORD-3 because the HMD LTs extend up to 110, while CONCORD’s LTs only up to age 99. This means that a lower proportion of the oldest patients who were followed up are included in SURVMARK’s computations (Fig. 1). At the same time, SURVMARK-2 has a disadvantage with the death certificate-only cases, as it includes an additional group of patients with unfavorable survival. However, Arnold et al. note that including death certificate-only and autopsy cases did not alter the overall patterns across countries [2]. These technical features play a smaller, but sometimes significant, role in the assessment of survival differences between countries. Albeit both studies worked from the same data frame between 2010 and 2014, analysis of the data points which were available in both analyses revealed differences in survival estimates ranging from − 1.9% point to + 4.3% point. The largest survival discrepancy in favor of CONCORD-3 was observed for colon tumors in New Zealand, while the greatest difference in favor of SURVMARK-2 was seen for rectal cancer in Denmark. Overall, among the 49 common localizations analyzed across countries, CONCORD-3 reported higher survival in 12 cases, one showed no difference, and SURVMARK-2 had higher survival in 36 cases.

Keeping all this in mind, attention must be drawn to the most important fact that variations in coding systems, classification methods, and cancer registration practices across countries can generate the largest survival differences, and for this reason these discrepancies are the primary potential challenges to international population-based comparisons [2]. Consequently, only the adoption of standardized registration, classification, and coding procedures, along with the application of more transparent and publicly accessible data processing methods could ensure more reliable international comparisons in cancer survival.

Important limitations of this study are firstly the small number of tumor localizations examined, which narrows the applicability of the findings. Nevertheless, instructive conclusions can be drawn because the tumor types studied have significantly different prognoses. Secondly, potential data quality issues, such as the proportion of death certificate-only (DCO) and morphologically verified percentage (MV%) cases which could introduce bias into the survival estimates. While DCO cases were excluded from our calculations due to their unknown survival times, cases were included which had no morphological verification. Higher MV% is associated with more accurate survival probabilities, while lower MV% often includes clinically or radiologically diagnosed cases, which may overestimate survival if non-cancer cases are misclassified as cancer or underestimate survival if advanced, late-stage cases are diagnosed clinically and included. In our study, ovarian tumors were most affected from this perspective, of which only 42.7% were morphologically verified. However, survival of cervical and breast cancer may be more accurate than this, whereas the MV% of both were above 70%. In summary, we have concluded that establishing an international consensus on the methodology of survival analysis is imperative. This includes defining the applicable databases for LT calculations, as well as determining main parameters for fine-tuning such as κ, λ and exact locations of knots.

## Electronic supplementary material

Below is the link to the electronic supplementary material.

Supplementary Material 1

## Data Availability

Requests for data sharing with research proposal should be addressed to Mr. István Kenessey, Head of the Hungarian National Cancer Registry, who will determine when, for, how long, for which specific purposes and under which conditions the requested data can be made available, subject to ethical consent.
