# Supplementary material for: The impact of life tables on age standardized net survival of real-life example databases
Source: BMC Med Res Methodol. 2025 May 26;25:145. doi: 10.1186/s12874-025-02600-7 (PMC12107785; doi:10.1186/s12874-025-02600-7)
Supplement: Supplementary file 1 — Supplementary Material 1 [file 12874_2025_2600_MOESM1_ESM.docx]

**Supplemental Material**

**Glossary of terms, abbreviations and acronyms**

**Age-standardized net survival**

**probability** Summary measure in cancer epidemiology that adjusts the net survival across different age groups to account for variations in the age distribution of cancer patients. It allows fair comparisons of survival probabilites between populations, regions, time periods, or demographic groups, by removing the effect of different age structures. Net survival is the probability that a person diagnosed with cancer would survive the disease, assuming that no other causes of death exist. It isolates cancer-specific survival from all-cause survival.

**CI** Confidence interval

**CONCORD-EWBANK** Ewbank relational model with parameters - is a reducible four-parameter system for modelling life tables. This method utilizes unsmoothed mortality rates in the same single age breakdown to derive a smoothed mortality profile for the given population.

**CONCORD-MFM** CONCORD Multivariable Flexible Model - Flexible Poisson model to derive the required fitted age- and sex specific mortality rates.

**CONCORD research program** Global initiative that monitors population-based cancer survival trends to evaluate the effectiveness of health systems in managing cancer. By analyzing survival data from cancer registries worldwide, CONCORD provides insights into disparities in cancer outcomes and informs cancer control policies.

**DCO** Cancer cases which can only be revealed from the death certificate (death certificate-only).

**EUROCARE-5** Fifth iteration of the EUROCARE (European Cancer Registry-based study on survival and care of cancer patients) project, which aims to monitor and analyze cancer survival probabilities across Europe. This comprehensive study provides insights into the effectiveness of cancer care systems by comparing survival outcomes across different countries and regions.

**Ewbank model** Ewbank relational model with parameters. This procedure uses four parameters. Two of these are according to the methodology of linear regression based on the Brass relational logit model:

**α parameter** describes the level of mortality,

**β parameter** characterizes the strength of the relationship between the standard and observed data.

**κ parameter** for younger ages,

**λ parameter** for older ages.

**GLOBOCAN, GCO** The Global Cancer Observatory (GCO) is an interactive web-based platform presenting global cancer statistics to inform cancer control and research.

**HCSO** Hungarian Central Statistical Office

**HLD** Human Life-Table Database

**HMD** Human Mortality Database

**HNCR** Hungarian National Cancer Registry

**IARC** International Agency for Research on Cancer

**LT** Life table. Statistical tool used in demography and epidemiology that summarizes the mortality experience of a population during a specific time period—typically a single calendar year. It estimates how long a hypothetical group of individuals would live if they were subject to the age-specific mortality rates observed during that period.

**lx column** Number of people surviving to exact age x.

**qx column** Probability of dying between age x and x+n.

**MV%** Proportion of registered tumors that were confirmed by histological examination (morphologically verified).

**NHIF** National Health Insurance Fund of Hungary

**Overall survival probability** Proportion of patients who are still alive during a certain time interval after their diagnosis with cancer, regardless of the cause of death.

**Pohar Perme method** Statistical approach to estimate net survival in cancer epidemiology, especially useful in population-based survival studies. It was introduced by Pohar Perme, Stare, and Estève in 2012 and has become the gold standard for unbiased net survival estimation.

**Restricted cubic spline** Flexible tool used in regression analysis — especially in medical and epidemiological research — to model non-linear relationships between a continuous predictor and an outcome variable, while maintaining smoothness and interpretability.

**Cubic spline** A piecewise-defined function made of cubic polynomials.

**Restricted** It imposes linear constraints at the boundary knots to avoid erratic behavior at the edges of the data range.

**Knots** Specific values of the predictor variable where the polynomial pieces connect.

**SURVCAN-3** Third phase of an international initiative led by the International Agency for Research on Cancer (IARC). Launched in September 2016, SURVCAN-3 aims to produce reliable and comparable cancer survival statistics in low- and middle-income countries (LMICs), particularly in Africa, Asia, and Latin America. The project seeks to benchmark national and subnational estimates of cancer survival to aid in the assessment of national cancer control policies.

**SURVMARK-2** Second phase of the International Cancer Benchmarking Partnership (ICBP), a global collaboration led by the International Agency for Research on Cancer (IARC). The project aims to measure and compare cancer survival, incidence, and mortality across seven high-income countries: Australia, Canada, Denmark, Ireland, New Zealand, Norway, and the United Kingdom.
